# Toward the standardization of radiopharmaceutical therapies: a technical note evaluating a clinical dosimetry workflow for single-time-point ^177^Lu SPECT/CT-based therapies

**DOI:** 10.1186/s40658-025-00764-1

**Published:** 2025-08-14

**Authors:** Taehyung Peter Kim, Wendy Siman, Vivek Mishra, Santiago Aguirre, Siju C. George

**Affiliations:** 1 Department of Radiation Oncology, Miami Cancer Institute, Miami, FL USA; 2 Carle Illinois College of Medicine, University of Illinois Urbana-Champaign, Urbana, IL USA; 3 Department of Radiology, University of Colorado School of Medicine, Aurora, CO USA; 4 Department of Medical Physics, Georgia Institute of Technology, Atlanta, GA USA; 5 Herbert Wertheim College of Medicine, Florida International University, Miami, USA

**Keywords:** Radiopharmaceutical therapies, Theranostics, $^{177}$Lu, Dosimetry, Treatment planning, Standardization

## Abstract

**Purpose:**

The lack of standardized dosimetry workflows in lutetium-177 ($^{177}$Lu) radiopharmaceutical therapies results in inconsistent absorbed doses and limits treatment planning. This study aims to evaluate the accuracy and variability of a single-time-point ^177^Lu SPECT/CT commercial workflow to help harmonize its protocol.

**Methods:**

The dosimetry workflow evaluated in this study predominatly followed that of MIM SurePlan^TM^ MRT. ^177^Lu SPECT/CT images of a Jaszczak and a NEMA phantoms were acquired in GE 670 DR scanner. Absorbed dose (Gy/MBq/s) was calculated in the background and sphere inserts with varied reconstruction iterations, calibrations, voxel-based dosimetry methods, and target volume segmentations. Ground truth absorbed doses were created using CT images and voxel S-value (VSV) water kernels. The validity of the density-corrected (DC) kernel for use in ground-truth dosimetry evaluations was further investigated. The accuracy and variability of the dosimetry workflow were evaluated using percent error and the coefficient of variation (CV) of mean absorbed doses.

**Results:**

Mean absorbed dose accuracy improved for both the voxel-based VSV and local deposition (LD) methods until 480 equivalent iterations for all target volumes. DC kernel was found viable for creating reference absorbed doses. The calibration CV was 5.18% when phantom and calibration regions were varied. The VSV method demonstrated absorbed doses that were 10 to 150% higher than those calculated with the LD method. The overall variability in absorbed dose reached up to 84% when reconstruction, calibration, dosimetry, and segmentation methods were varied.

**Conclusions:**

A single dosimetry workflow has demonstrated markedly large variability in absorbed dose accuracy. By evaluating the accuracy of absorbed dose, our study helped to propose a harmonized MIM SurePlan^TM^ MRT workflow for single-time-point $^{177}$Lu SPECT/CT-based therapies.

## Introduction

Radiopharmaceutical therapies (RPTs) are a form of radiation therapy that use radionuclide-labelled agents to deliver targeted irradiation of tumor cells either systemically or locoregionally. Lutetium-177 ($^{177}$Lu) is a recent addition to the rapidly growing arsenal of RPTs with European Medicines Agency or US Food and Drug Administration approval [1]. $^{177}$Lu agents include $^{177}$Lu-DOTATATE, which targets neuroendocrine tumors via somatostatin receptors, and $^{177}$Lu-labeled prostate-specific membrane antigen (PSMA), which is used to treat metastatic castration-resistant prostate cancer [2, 3].

Part of the perceived therapeutic potential of $^{177}$Lu-based therapies lies in personalized treatment planning that use dosimetry [4]. In this regard, LUMEN and LUTADOSE trials have demonstrated dose-response relationships with progression-free survival outcomes, highlighting the benefit of post-cycle dosimetry [5, 6]. Ongoing research has focused on characterizing post-treatment imaging protocols and establishing individualized treatment plans [7, 8]. In addition, significant effort into the application of single-time-point imaging is underway in an attempt to reduce the number of patient visits associated with serial imaging [9, 10].

Clinically, treatment plans for RPTs are implemented using commercially available softwares [11]. While these platforms provide a framework for treatment planning, they offer limited guidance on protocol implementation, often requiring arbitrary parameter decisions that contribute to significant variability between inter- and intra-dosimetry workflows [12, 13]. Such variability directly affects dose calculations, limits the clinical utility of dosimetry, and has underscored the need to harmonize dosimetry workflows [10, 14, 15].

One potential approach to achieving harmonization among different commercially available workflows is to systematically evaluate their individual performances, enabling the characterization and quantification of workflow-specific differences. Understanding these differences is essential for establishing reliable methods for translating or interpreting dose metrics across various platforms. Such understanding will improve patient management by promoting the comparability of dose-response relationships and, in turn, ensuring consistent patient outcomes. As an initial step toward this objective, the present study assessed the end-to-end accuracy and variability of the MIM SurePlan$^{\textrm{TM}}$ MRT dosimetry workflow for single-time-point $^{177}$Lu SPECT/CT-based therapies. In turn, this evaluation aimed to support the harmonization of one clinical dosimetry workflow.

## Materials and methods

### Overview of evaluation framework

**Fig. 1:**
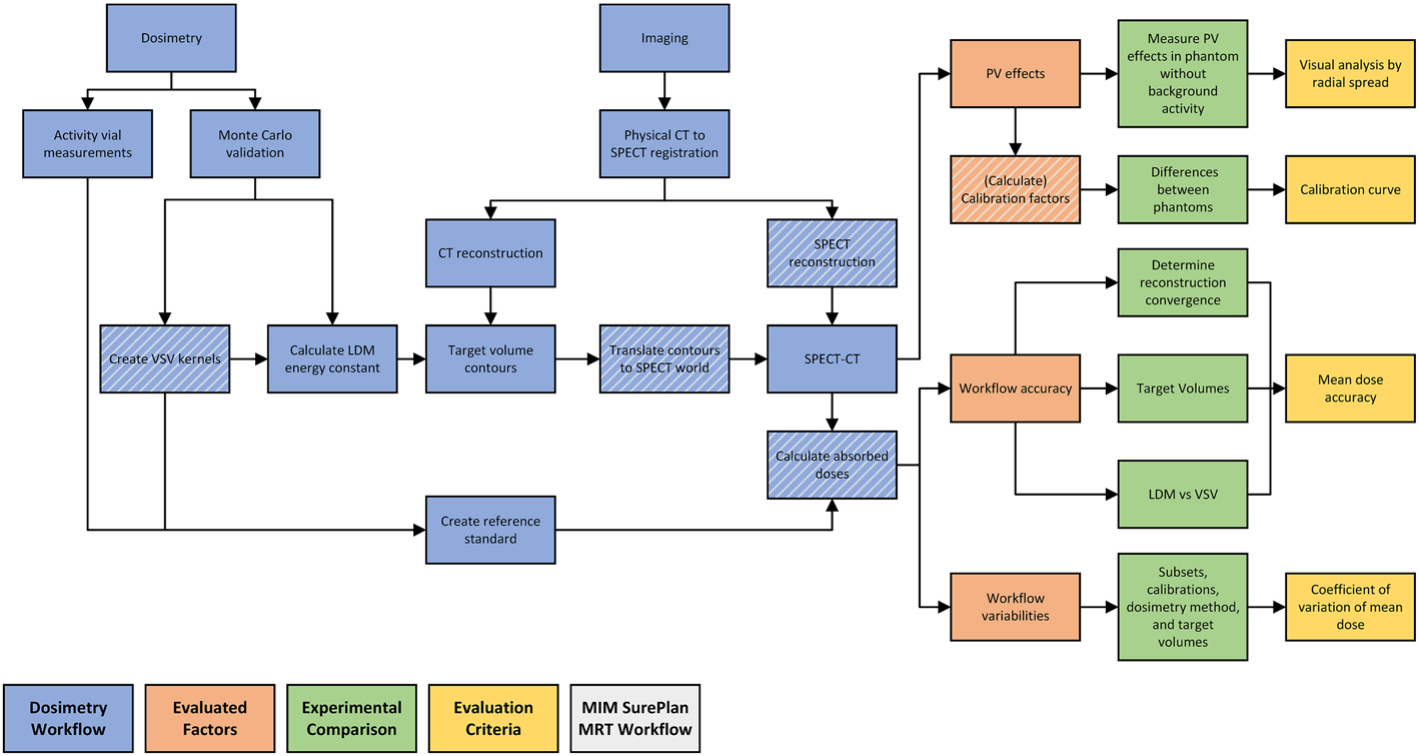
A summary of the clinical dosimetry protocol and evaluations performed in this study are illustrated. Dashed boxes denote the components shared between MIM SurePlan^TM^ MRT and the study's full dosimetry workflow. LDM denotes the LD method

The MIM SurePlan^TM^ MRT workflow for singe-time-point SPECT/CT dosimetry, subsequently referred to as "MRT", was evaluated in this study. The MRT workflow includes SPECT/CT image reconstruction, calibration procedures, time-integrated activity (TIA) calculations, and absorbed dose estimation using the voxel S-value (VSV) method. In this study, the MRT workflow was independently implemented to allow for the evaluation of the accuracy and variability of its individual components. The local deposition (LD) method was additionally evaluated for completeness, while TIA evaluations were excluded. The dosimetry workflow implemented and evaluated in this study was summarized in Fig. 1, with details provided in the following sections.

### $^{177}$ Lu SPECT/CT protocol

This study utilized a singular Discovery SPECT/CT system, GE 670DR (GE Healthcare, Cleveland, USA), with a parallel-hole, medium energy, general purpose collimator and a 3/8” crystal thickness. SPECT data was acquired with a primary energy window of 187-229 keV and two scatter windows of 166-187 keV and 229-250 keV. The physical registration correction between the CT and SPECT scanners was previously reported [16].

All quantitative SPECT/CT reconstructions were performed using ordered-subset expectation maximization (OSEM) with user-defined subsets and iterations. This study reconstructed images using a range of 24 to 480 equivalent iterations, applying five different subsets (1, 4, 8, 12, 24) at each 24-iteration interval, resulting in a total of 100 reconstructed images. A configuration with 1 subset and 192 iterations was referred to as ‘1s192i,’ where ‘s’ indicates the subset and ‘i’ indicates the iteration.

SPECT/CT images were reconstructed with attenuation correction based on CT, triple energy window (TEW) scatter correction, decay correction, and a geometric component of the collimator detector response (CDR). All resulting images had an isotropic voxel size of 4.42 mm, and no post-image filtering was applied. The CT was reconstructed with a filter back projection and a body filter. The CT image had a voxel size of 0.98 x 0.98 x 3.75 mm. A bilinear curve was utilized to determine attenuation coefficients by scanning a phantom with known density inserts.

### Experimental procedure

**Fig. 2:**
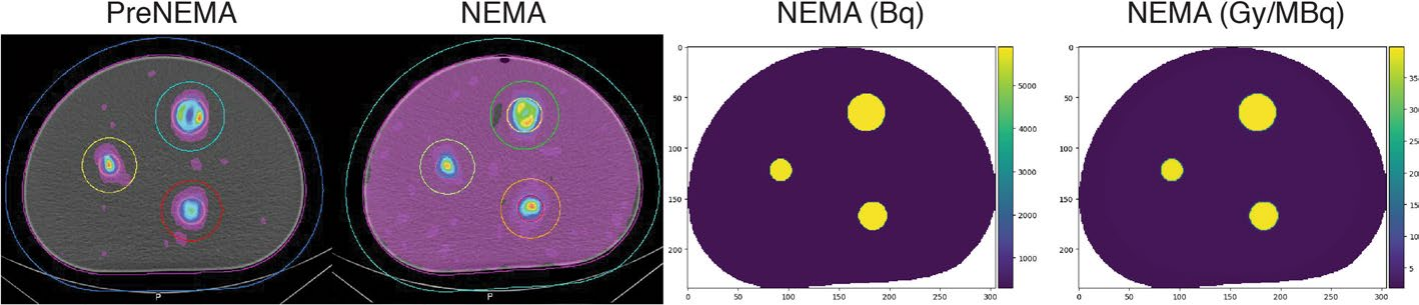
The reconstructed NEMA phantom (1s192i) are illustrated with their corresponding contoured volumes visualized to the left. On the right, an example of the activity and absorbed dose ground truth NEMA phantoms are illustrated

This study utilized a Jaszczak and NEMA phantom with three SPECT/CT scanning sessions. For the first set of scans, the Jaszczak phantom body (labelled JASZ) was filled with approximately 790 MBq of $^{177}$Lu per vendor’s instructions to investigate the standard method for SPECT/CT calibration.

A modified NEMA phantom was then used to obtain two additional scans. In the first scan (labelled PreNEMA), three spherical inserts with diameters of 37 mm (26.5 mL), 28 mm (11.5 mL), and 22 mm (5.57 mL) were filled with 71.7 MBq of $^{177}$Lu and an ethylenediaminetetraacetic acid (EDTA) solution to prevent adhesion to the sphere walls. The spheres were positioned equidistantly in the phantom to minimize overlapping partial volume (PV) effects in the reconstructed image. The background contained solely EDTA solution. In the second scan (labelled NEMA), the phantom was filled with $^{177}$Lu to achieve a mean sphere-to-background or signal-to-background ratio (SBR) of approximately 10:1 using 940 MBq of total activity.

### Target volumes

Three target volumes were defined for each of the 37 mm, 28 mm, and 22 mm sphere inserts: (1) the spherical inserts alone (spheres), (2) the PV volume (PVV), and (3) the spherical inserts plus the PVV (sphere plus PVV). A whole-body region, corresponding to its target volume, was additionally defined.  PV effects were visually assessed using PreNEMA phantom images reconstructed with one subset across 24 to 480 equivalent iterations. To estimate PV effects, object contours were radially expanded to include all visible counts in the SPECT image displayed at maximum intensity. The surrounding shell volume containing the counts solely attributed to PV effects was defined as the PVV. The whole-body target volume was defined as the volume within the phantom body that excluded the spheres plus PVV.

Target volumes were contoured on the CT dataset with the help of auto-contouring tools using MRT. The whole body contour tool was used to first automatically detect the boundaries of the phantom’s body and corrected manually in the boundaries of the axial slices. Spherical volume tools were utilized to create spheres based on the specified manufacturing diameters, which were positioned using both coronal and axial views.

**Fig. 3:**
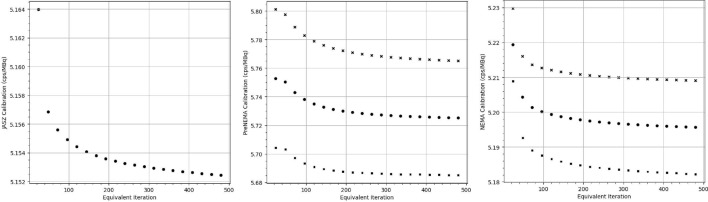
Calibration factors for all phantoms reconstructed with 1s24i to 1s480i and their positive (crosses) and negative (squares) 95% confidence intervals were illustrated. The JASZ phantom was reconstructed solely with the MLEM algorithm

**Fig. 4:**
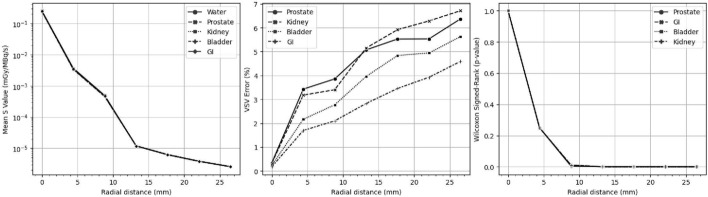
VSV kernels of (left) five different tissues and (middle) the percent errors between the density corrected (DC) kernel and other tissue kernels were summarized for radial distances of 4.42 mm. The (right) impact of the error at each radial distance was illustrated using p-values

**Fig. 5:**
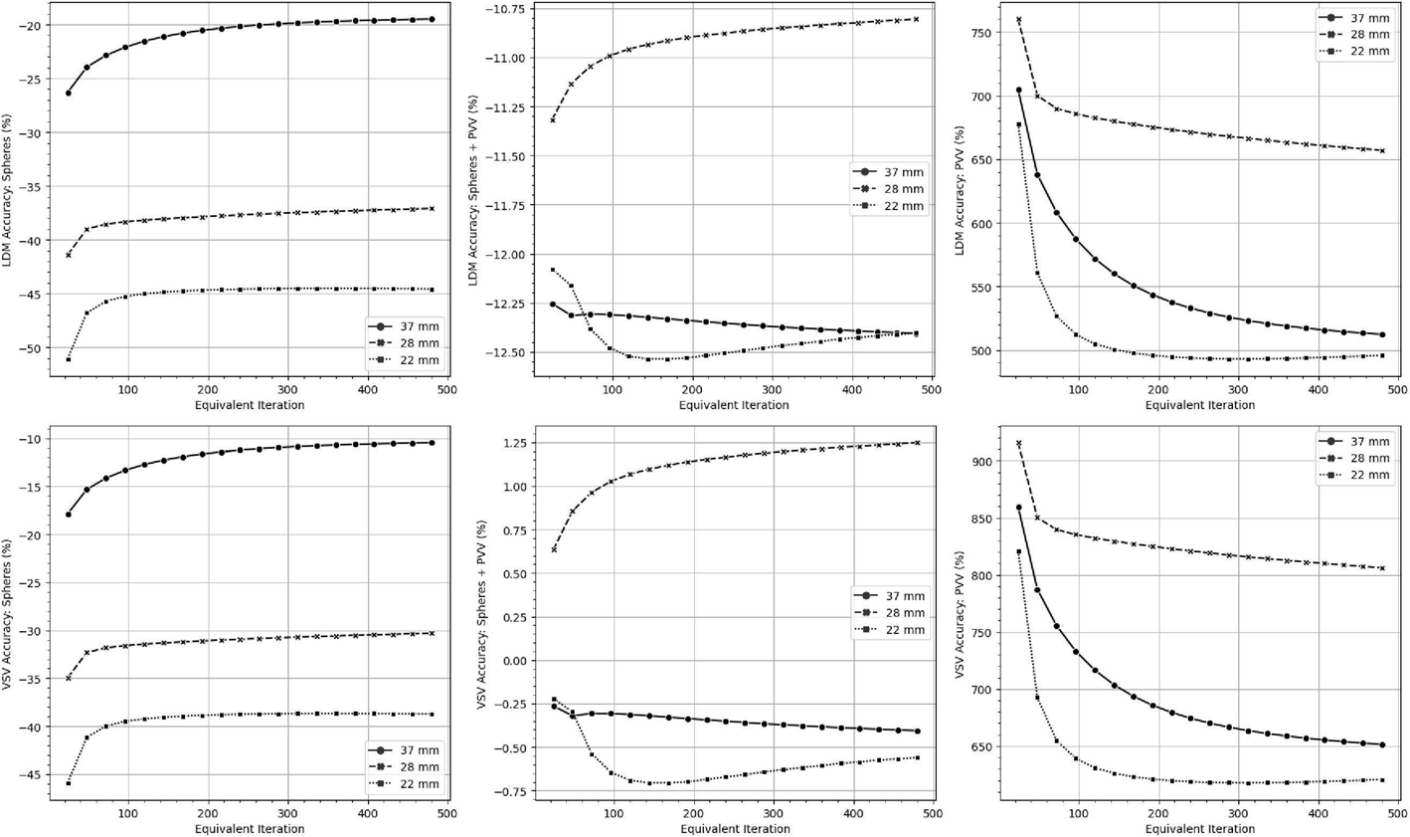
The mean dose accuracy for the PreNEMA phantom is illustrated for different dosimetry methods, with target volumes indicated on the y-axis. LDM denotes the LD method

### Calibration factors

Calibration factors (CF) that converts counts to activity (Bq) with activity calibrations are calculated in MRT using Eq. 1. This equation uses the total counts per second in a specific volume ($c_{map}$) with *N* voxels, the activity administered to the same volume ($A_{0}$), the number of projections (*P*), and the duration of each projection scan ($T_{p}$).

1 $$\begin{aligned} CF \; (cps/Bq)= \frac{\sum \limits _{n=1}^{N}c_{map} \, (counts)}{A_{0} \, (Bq) *P*T_{p} \, (s)} \end{aligned}$$

The volumes of calibration were considered the regions where the activity was known, which were then expanded by the PVV. These regions represented the entire body phantom for the JASZ and NEMA phantoms or the sphere target volumes for the PreNEMA phantom.

The variability in calibration factors was evaluated using percentage-based coefficients of variation (CVs). A CV$_{act}$ was calculated to represent the CV in calibration methods across all subsets and iterations within each phantom, while CV$_{cal}$ incorporated both calibration and inter-phantom differences.

### Reference standard

Ground truth dose distributions (Fig. 2) were generated from a CT scan, with activity experimentally determined using a dose calibrator (Figure S1). It was assumed that activity was homogeneously distributed within the target volumes and background. Absorbed dose calculations were performed using target volumes defined in the CT coordinate system. reDoseMC, previously validated for its accuracy and workflow, was used to generate VSV kernels for various soft tissues matching the CT coordinate system. These kernels were then convolved with the ground-truth image to calculate reference absorbed doses [17].

To extend the generalizability of the reference standard for future $^{177}$Lu evaluations, this study assessed the accuracy of density-corrected (DC) kernels for absorbed dose calculations by simulating and comparing five different VSV soft-tissue equivalents derived from ICRU Report 46: gastrointestinal (GI), prostate, soft tissue, kidney, and bladder [18]. The key simulation parameters for all Monte Carlo simulations, including the VSV kernels, were summarized in Supplemental Table S1. Differences between the DC and other soft-tissue kernels were determined by plotting radial dose profiles and calculating relative percent errors at each radial distance, i.e., (VSV kernel − DC kernel)/(DC kernel) [17]. The statistical impact of DC kernel errors was evaluated by comparing voxel values contained within each radial distance using the Wilcoxon signed-rank test.

### Dosimetry evaluations

MRT currently performs ^177^Lu dosimetry exclusively using the VSV method with a 3 mm isotropic voxel-size kernel. However, both the LD and the VSV method can be applied for voxel-based dosimetry [14]. In this study, pyreDose and reDoseMC were used to implement both these methods [17, 19]. To ensure clinical relevance, the comparability between the VSV kernels used in MRT and those in reDoseMC was further analyzed and shown in Supplemental Figure S2 [20, 21].

All SPECT/CT dose estimates were calculated based on the SPECT coordinate system and performed by self-calibration, unless otherwise noted. When performing the LD method, an average energy of 0.134 MeV was utilized. Normalized absorbed dose by activity (Gy/MBq/s), subsequently referred to as absorbed dose, was utilized to permit a generalized analysis of absorbed dose accuracy and variability without the need for TIA calculations.

The end-to-end evaluation of accuracy for the single-time-point $^{177}$Lu treatment protocol was assessed using percent error and mean dose differences between voxel-based methods and the ground-truth absorbed dose. Absorbed dose accuracy was evaluated based on images reconstructed with one subset, as these images represented the most accurate reconstructions due to demonstrated convergence [22, 23].

The impact of workflow variability (CV$_{dose}$) was analyzed by evaluating changes in reconstruction subsets and iterations, self-calibrations, and dosimetry methods, all measured within the same equivalent iteration, target volume, and phantom. Total variability (CV$_{total}$) expanded upon the CV$_{dose}$ metric by incorporating the JASZ calibration and different target volumes for each sphere insert to assess the overall variability of a single dosimetry workflow within the same phantom. For CV$_{total}$, the target volumes examined included the sphere and the sphere plus PVV.

## Results

**Fig. 6:**
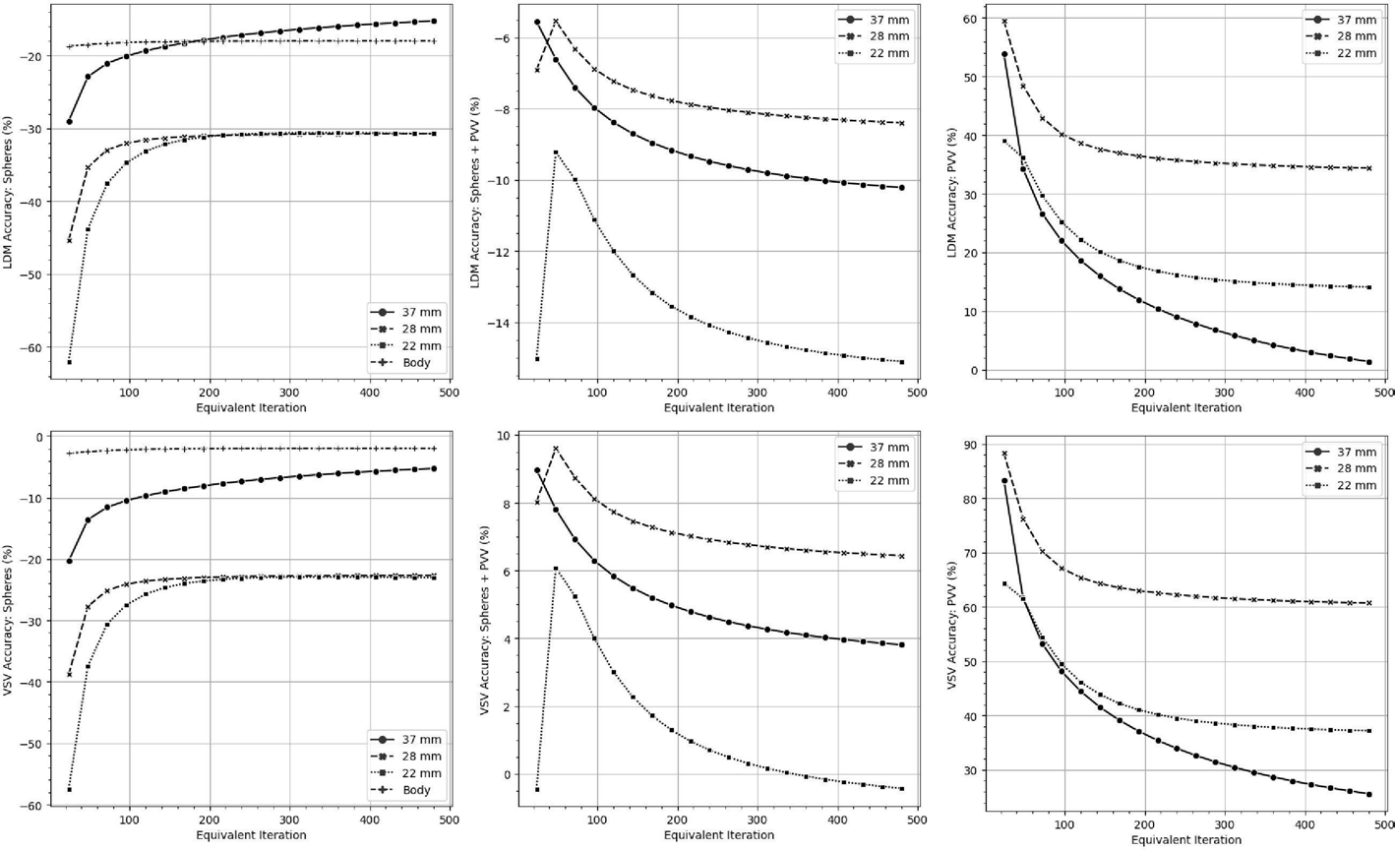
The mean dose accuracy for the NEMA phantom is illustrated for different dosimetry methods, with target volumes indicated on the y-axis. LDM denotes the LD method

**Fig. 7:**
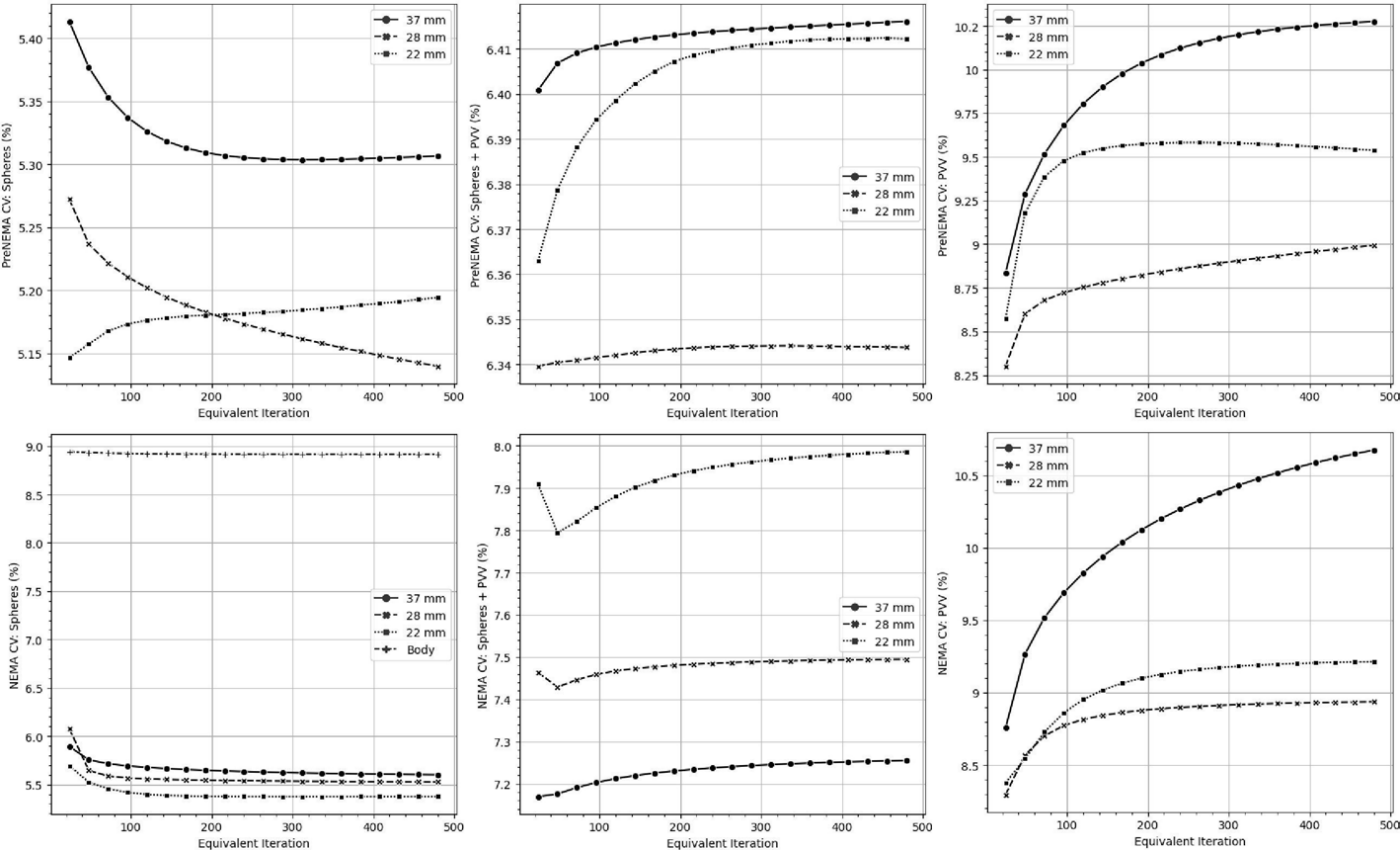
The percentage-based coefficient of variation (CV) for the entire dosimetry workflow, including reconstruction algorithm, calibration, and dosimetry method, was summarized for both the PreNEMA and NEMA phantoms across different equivalent iterations and target volumes, as shown on the y-axis

### Partial volume estimations

Figure 2 visualized the PreNEMA, NEMA, and ground truth phantoms. The PreNEMA phantom was filled with activity in the sphere target volumes; however, this activity was found to be spread outside its anatomical volume, extending approximately 18 mm from the spheres. This PV radius extension was consistent across both the sphere target volumes and the background volume from 24 to 480 iterations in the PreNEMA images. Furthermore, the sphere target volumes in both the PreNEMA and NEMA phantoms exhibited non-homogeneous activity, particularly in the larger sphere inserts.

### Calibration factors

Figure 3 illustrated the mean calibration from images reconstructed with one subset, along with two standard deviations from the mean. When compared among the same phantom, the CV$_{act}$ was lower than 0.05%; the PreNEMA and NEMA CV$_{act}$ were 0.37% and 0.15%, respectively. There was a CV$_{cal}$ of 5.18% between calibration factors of all phantoms and reconstructions.

### Soft-tissue VSV kernels

Figure 4 illustrated the soft-tissue VSV kernels, its mean error between the DC kernel and other tissue materials at each radial distance, as well as their significance. The prostate, kidney, bladder, and GI tract showed voxel percent differences ranging from 0.18–0.34% at the source to 1.70–3.43%, 2.10–3.86%, 2.83–5.15%, 3.46–5.92%, 3.93–6.28%, and 4.59–6.37% at increasing radial distances. With a significance level set at 0.05, significant differences were observed between the DC and all other soft-tissue kernels beginning at approximately an 8.84 mm radial distance.

### Evaluating dose accuracy

Figures 5 and 6 visualized the mean dose accuracy of the LD and VSV method for the PreNEMA and NEMA phantoms reconstructed with one subset and self-calibrated to their equivalent iteration. The sphere target volumes of the Body, 28 mm and 22 mm spheres, regardless of the dosimetry method, were found to converge in accuracy at around 1s192i while the accuracy of the 37 mm sphere increased until 1s480i. The mean absorbed dose accuracies for target volumes that included PV regions were found to converge around 1s192i, while the accuracies for PVVs began to converge at 1s480i. The exact percent errors  were summarized in Supplemental Table S3 and S4.

When considering both phantoms, the LD method consistently calculated lower absorbed doses than the VSV method across all sphere target volumes, with differences ranging from 5.9% to 10%. Accuracy improved when PVVs were included in the target volume for both the VSV and LD methods reducing errors to ≤6.5%. However, target volumes consisting solely of PVVs showed significant overestimation of absorbed doses.

Figure 7 illustrated the CV$_{dose}$ of mean doses when implementing different subsets, dosimetry method, and target volumes. For both the PreNEMA and NEMA phantom, the CV$_{dose}$ generally decreased with increasing iteration for the sphere target volumes. The CV$_{dose}$ increased and then plateaued for target volumes including the PVV, with a maximum below 10.7%. When implementation differences included factors of calibration, reconstruction, dosimetry methods, and target volumes, the CV$_{total}$ ranged from 74.4% to 83.7% for the PreNEMA phantom and 66.5% to 75.8% for the NEMA phantom.

## Discussion

This study aimed to systematically evaluate the end-to-end accuracy and variability of MRT’s single-time-point $^{177}$ Lu SPECT/CT workflow. Whereas prior studies focused on variability, this study primarily assessed the accuracy of MRT’s $^{177}$Lu clinical dosimetry workflow to characterize individual workflow differences and propose a harmonized protocol [14, 24].

Currently, a standardized evaluation method is not yet established for RPTs. As a result, we adapted a framework from the field of other beta-emitters, particularly trans-arterial radioembolization (TARE), which shares many imaging and dosimetry principles with $^{177}$Lu therapies [25]. A key prerequisite for dosimetry evaluations of RPTs is the implementation of a robust reference standard and appropriate dosimetry evaluations [26]. In this study, the dose kernels required for dosimetry using the VSV method were first generated using a previously validated Monte Carlo software, then compared against those utilized by MRT to ensure its clinical comparability (Figure S2) [17]. Of note, a modified NEMA phantom and mean dose estimates were used to assess the dosimetry workflow accuracy. Although voxel-based metrics, such as dose-volume histograms, can be calculated, the reconstructed images (Fig. 2) lacked sufficient homogeneity within the sphere inserts, even with the addition of EDTA solution. These effects may be attributed to artifacts from non-circular acquisitions. However, these variations may also reflect the true differences in activity distribution within the sphere inserts of our phantom. Because it was not possible to experimentally confirm activity homogeneity within the inserts, reference dose calculations were based on mean doses, which are less sensitive to inhomogeneous activity distributions [27].

Pertinently, this study investigated the differences between the DC kernel and other soft-tissue kernels. Differences were found to be non-significant up to a radial distance of 4.42 mm, with significant differences observed beyond that point. The increased error in absorbed dose calculations for voxels at greater radial distances was expected due to the combined effects of reduced secondary particle production and the limited number of simulated histories used to generate the VSV kernel. However, these statistically significant differences likely had minimal impact, as they occurred in kernel voxels where dose values were several orders of magnitude lower than those in the source voxel. These findings support the use of the DC kernel for ground truth dose calculations and further reinforce its viability for clinical applications [18].

One surprising finding of our study was that the VSV method in $^{177}$Lu SPECT/CT dosimetry resulted in larger absorbed doses than the LD method. Extensive research on TARE dosimetry has shown that the LD method provides dose estimates of greater magnitude and accuracy, as it effectively simulates beta energy transport when combined with the limited spatial resolution of SPECT or PET imaging systems [28]. Notably, our study demonstrated that absorbed doses calculated with the VSV method were between 10% to 150% higher than those calculated with the LD method depending on the target volume. This finding suggests that secondary particle contributions in $^{177}$Lu therapies have a greater impact on absorbed dose estimates than those in TARE therapies [17]. More broadly, it highlights that the same dosimetry method can yield different effects even within the similar category of radiopharmaceuticals (i.e., beta-emitters).

Another unique finding of this study was the investigation of dose accuracy for target volumes that encompassed the entire image. These target volumes included the spheres combined with PVVs that simulated SPECT segmentation methods, PVVs that measured artificial dose spillover, and whole-body target volumes. We observed that while sphere dose accuracy generally converged at 192 iterations, all target volumes required 480 iterations to achieve convergence. This slower convergence was attributed to the additional iterations required to reach maximum scatter and CDR corrections. This observation has direct clinical consequences, best illustrated by the standard example in which a sphere represents the tumor, and the surrounding area denotes normal tissue or background. In such cases, segmentation methods that employ SPECT thresholding of the tumor volume (i.e., Sphere + PVV) are likely to yield more accurate mean doses than anatomical contouring. However, the thresholding segmentation technique relies on activity gradients that are likely to be variable when considering incomplete scatter and CDR corrections. In contrast, normal tissues that include tumor margins (i.e., PVV) will overestimate the mean dose. These results suggest that reconstructing to convergence, or its equivalent iteration, is warranted to achieve more stable dose estimates. Otherwise, these findings highlight the urgent need to address the spatial limitations inherent in $^{177}$Lu SPECT/CT that underlie these issues, with potential solutions involving deep learning techniques [29].

This study further assessed the variability within a single dosimetry workflow. In this regard, the CV_*cal*_ was 5.18% when calibrations were based on different phantom setups. For a single dosimetry workflow, the CV$_{dose}$ rose to 10.7% when the reconstruction subset was varied, which, by extension, included changes in self-calibration and dosimetry methods (Fig. 7). Notably, we observed a CV$_{total}$ of approximately 84% when all parameters within a single dosimetry protocol were altered, even within a workflow using the same SPECT/CT scanner [24]. These results empirically confirmed that even minor hyperparameters, such as the OSEM algorithm subset, can affect absorbed dose consistency (Fig. 7). These findings aligned with prior studies on calibration and single-workflow variability, emphasizing the critical need to harmonize every parameter within a single clinical dosimetry workflow [15, 17, 30].

By assessing the end-to-end accuracy and variability of different parameters within a single workflow, this study captures the evaluation of the major treatment protocol combinations that may be employed in MRT’s single-time-point $^{177}$Lu SPECT/CT workflow. It is worth noting that TIA evaluations require well-established ground truth pharmacokinetics data specific to either $^{177}$Lu-DOTATATE or $^{177}$Lu-PSMA therapies [31, 32]. As such, in the absence of an established standard, complete evaluations of clinical workflow accuracy remain infeasible. To address this, our study implemented normalized absorbed doses (Gy/MBq/s) to enable quantitative workflow evaluations. Consequently, an ideal MRT workflow for $^{177}$Lu SPECT/CT-based therapies may be derived from the results of this study, though some decisions may be considered debatable. Accordingly, we recommend using OSEM-reconstructed images with a fixed 480 equivalent iterations, comprising 20 iterations and 24 subsets. This combination helps balance accuracy and speed, ensuring consistent absorbed dose calculations across all target volumes. Calibration remains a complex issue; large calibration variability was observed when either or both the phantom and calibration regions were changed. These results highlight the fact that consistency in calibration methodology is likely more critical than the specific method used. Furthermore, both the VSV and LD methods were considered viable for dosimetry, provided their dose characterizations are well understood. If a choice can be made, we recommend applying the LD method, as it produces more interpretable dose results and can be adjusted for PV effects. It further reduces the dose overestimation around the true volumes of $^{177}$Lu uptake. Worth noting is the direct correspondence of the LD method to organ-based MIRD dosimetry when homogeneously approximated densities are utilized [19]. Thus, the findings of this work pertaining to the LD method are also applicable to MIRD methods that may be utilized in $^{177}$Lu dosimetry.

To our knowledge, this study is the first to empirically evaluate the accuracy of ^177^Lu dosimetry workflows and to implement an initial evaluation framework. This novelty introduces several limitations. One limitation is the requirement for single-time-point $^{177}$Lu SPECT/CT-based therapies to establish a base image suitable for TIA implementation. It is generally recommended that phantoms replicate the SBR of base images that exhibit stable pharmacokinetics across patients [33]. This consideration is particularly relevant, as our study utilized normalized absorbed doses to enable evaluations of dose accuracy in the absence of established pharmacokinetic data. Normalized absorbed doses allow for the future incorporation of TIA accuracy once such data becomes available. However, it is unclear whether the SBR used in this study meets this criterion, which may affect the external validity of our findings. Nonetheless, if the same commercial software or workflow is used, different SBRs are expected to produce similar trends in absorbed dose accuracy, even if exact values may vary.

Another caveat of our study is that it employed a slightly modified version of MRT’s workflow. Typically, MRT uses a single VSV kernel with 3 mm isotropic voxel sizes for $^{177}$Lu dosimetry and trilinearly interpolates voxel sizes to match the VSV kernel. In contrast, this study generated VSV kernels that directly matched the SPECT voxel sizes, likely resulting in slightly improved dose accuracies. Additionally, the LD method was investigated in this study. Although not currently available within MRT’s $^{177}$Lu workflow, the LD method is supported by MRT for other isotopes and is a viable approach for $^{177}$Lu dosimetry [14]. Its inclusion in this study reflected its anticipated implementation.

Finally, future work is needed to assess the accuracy of different SBRs, co-registration methods, TIA calculations, and voxel-level absorbed dose accuracies. Our study used a modified NEMA phantom and mean dose to evaluate the dosimetry workflow’s accuracy. Although more anatomically representative phantoms, such as 3D-printed anthropomorphic phantoms, are available, they still rely on spherical inserts for dose evaluations, which can be adequately represented by the inserts in the NEMA phantom [34]. In other words, the spherical inserts may be considered the primary limiting factor in complex dose evaluations. Future studies may consider using phantoms with physical inserts better suited for evaluating the accuracy of voxel-based dosimetry [35].

## Conclusion

This study evaluated the single-time-point $^{177}$Lu SPECT/CT clinical dosimetry workflow of MIM SurePlan™ MRT. Convergence was achieved for all target volumes at 480 iterations. The use of DC kernels for ground-truth dose calculations was validated, supporting their clinical utility. Absorbed doses calculated with the LD method were generally lower than those calculated by the VSV method for all target volumes. A CV of up to 84% was observed within a single protocol when reconstruction algorithms, calibration, dosimetry methods, and target volume segmentation were modified. Overall, it is recommended that $^{177}$Lu protocols use OSEM images reconstructed with 24 subsets and 20 iterations, a single consistent phantom calibration, and the LD method for dosimetry.

## Additional file

Supplementary file 1 (pdf 2605 KB)

## Data Availability

The data that were generated and analyzed in this study are available from the corresponding author on reasonable request.
