# Supplementary material for: Toward the standardization of radiopharmaceutical therapies: a technical note evaluating a clinical dosimetry workflow for single-time-point 177Lu SPECT/CT-based therapies
Source: EJNMMI Phys. 2025 Aug 14;12:76. doi: 10.1186/s40658-025-00764-1 (PMC12354439; doi:10.1186/s40658-025-00764-1)
Supplement: Supplementary file 1 — (pdf 2605 KB) [file 40658_2025_764_MOESM1_ESM.pdf]

Toward standardization of radiopharmaceutical therapies: A technical note evaluating the clinical dosimetry workflow for single-time-point ^{177}Lu SPECT/CT protocols

Taehyung Peter Kim[1,2*], Wendy Siman[3], Vivek Mishra[1], Santiago Aguirre[1,4] and Siju C. George[1,5]

[1]Department of Radiation Oncology, Miami Cancer Institute, Miami, Florida, USA.
[2]Carle Illinois College of Medicine, University of Illinois Urbana-Champaign, Urbana, Illinois, USA.
[3]Department of Radiology, University of Colorado School of Medicine, Aurora, Colorado, USA.
[4]Department of Medical Physics, Georgia Institute of Technology, Atlanta, Georgia, USA.
[5]Herbert Weinstein College of Medicine, Florida International University, Miami, Florida, USA.

Supplemental

Dose calibrator measurements

Quantitative SPECT/CT imaging and dosimetry require precise measurements of administered activity. In this study, a ^{177}Lu source was supplied by Advanced Accelerator Applications^{TM} and measured with an ATOMLAB^{TM} 500 reentrant well chamber (dose calibrator) calibrated to a NIST traceable ^{177}Lu source vial. All activity measurements were time-stamped so that decay corrections could be applied based on the time of scan.

For accurate determination of activity transferred to a phantom, any residual activity remaining in the empty syringe was measured by the same dose calibrator. The impact of varying measurement geometry in the vial chamber was accounted by the following empirical technique. A series of activity extractions were made from the vial using the syringe from 2 - 18 mL. For each extraction, residual vial activity (difference between before and after vial extraction) provided the true activity in the syringe. The corresponding measured syringe activity was obtained and plotted against the true activity from vial measurements. This process was repeated for seven data points of net extracted activity, ranging from 25.9 MBq to 895 MBq and a fitted to a quadratic function as shown in Figure 1.

Before filling of the Jaszczak and NEMA phantoms, a 2.5 μM ethylenediaminetetraacetic acid (EDTA) solution in distilled water was mixed with ^{177}Lu to prevent the adhesion of ^{177}Lu molecules to the phantom walls. To maintain uniformity, the same batch of EDTA solution was used to fill the phantom in all the scans.

The comparison of ^{177}Lu VSV kernels

The ^{177}Lu beta spectrum from reDoseMC was benchmarked against experimental RadarDecay data (https://www.doseinfo-radar.com/RADARDecay.html). Additionally, a ^{177}Lu kernel was created using the same material and density composition as the previously published MCNP kernel, which set the material to water (ICRU Report 46) with a density of 1.0 g/cm^3. This comparison was used to validate substituting the kernel in MIM SurePlan with that from reDoseMC. All VSV kernels generated using reDoseMC placed a ^{177}Lu source in the center voxel of a cubic volume, simulated with the G4RadioactiveDecay physics inherent to Geant4. The VSV kernel simulations with reDoseMC utilized Penelope physics and the parameterized geometry of G4Nested. Mean radial doses were calculated by averaging the voxel doses at a radial distance of 4.42 mm.

Figure 2 illustrated the validation of the ^{177}Lu Monte Carlo beta spectrum and compares the VSV kernel generated by reDoseMC to the MCNP kernel used in MIM SurePlan^{TM} MRT. On the left side of Figure 2, the beta decay spectrum generated from reDoseMC is compared to experimental data. The beta decay spectrum shows a difference of less than 2.5% up to 0.4 MeV when the relative abundance percentage is reasonable, with an outlier observed at the highest energy bin. Additionally, the comparability between the VSV kernel generated with reDoseMC and the one used in MIM SurePlan^{TM} MRT, produced by MCNP, is presented. The comparisons of the radial dose profiles demonstrate similar results, with a maximum mean radial dose difference of 5.7%, supporting the validity of substituting reDoseMC VSV kernels for those used in MIM SurePlan^{TM} MRT.

Recovery coefficients

Recovery Coefficients (RCs) were used to initially characterize the SPECT/CT images. RC was calculated by dividing the imaged activity concentration by the actual activity concentration in the same volume and determined for both the PreNEMA and NEMA phantoms. The experimental phantom setup (A_{spect}) and the known activity concentration in each volume (A_{ref}), normalized by their corresponding volumes were used to calculate the RCs. Volume maps were determined either from SPECT images (V_{spect}) or low-dose CT images (V_{ref}). These recovery coefficients were obtained for the three spherical inserts and the body phantom.

$$RC\ (\%) = \frac{\frac{1}{X}\sum_{x=1}^{X} A_{spect}/V_{spect}\ (Bq/mL)}{\frac{1}{Y}\sum_{y=1}^{Y} A_{ref}/V_{ref}\ (Bq/mL)} * 100 \quad (1)$$

Figure 3 demonstrated the RCs for the PreNEMA and NEMA phantoms after performing MLEM reconstructions while Table S2 listed the RCs at 192 and 480 iterations. For both PreNEMA and NEMA phantoms, the RC plateaued at 192 iterations for all three sphere target volumes. The activity quantified remained relatively stable up to 480 iterations. In the PreNEMA phantom, greater activity was recovered as target volumes became larger, i.e. 37 mm. A similar trend was established in the NEMA phantom, except the 28 mm and 22 mm spheres activity recovery plateaued similarly at around 192 iterations. Generally, more activity was recovered in NEMA phantom, where activity was present in the background target volume.

Additional Tables

Additional information pertaining to the Monte Carlo simulation parameters, recovery coefficients, and absorbed dose calculations were summarized in Tables 1-5, respectively.

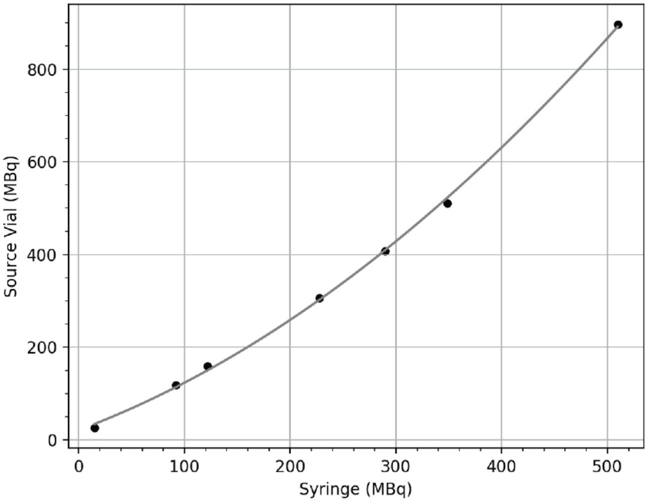

Fig. 1 Illustrates the fit utilized to convert source vial measurements with the syringes. Quadratic Fit Equation: $y = 0.06192x^2 + 0.855x + 0.5732$

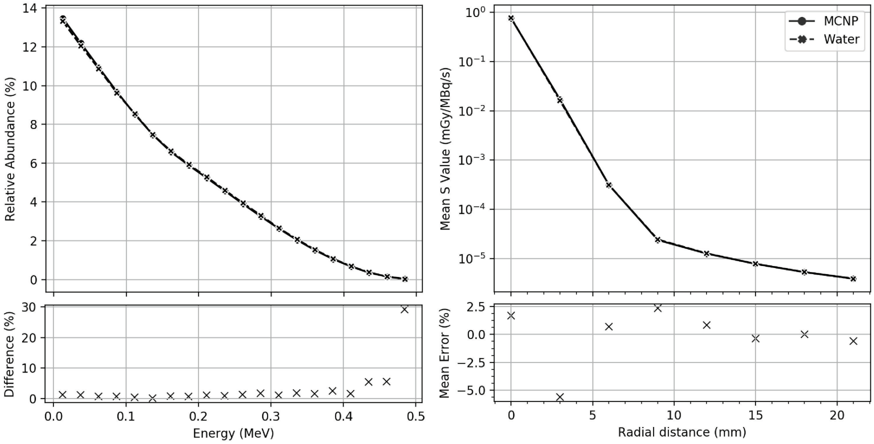

Fig. 2 The comparisons in the beta decay spectrum to experimental data (left), and the comparison between the ^{177}Lu VSV validation kernels (right).

Table 1 Details of Monte Carlo simulations

	Validation Kernel	CT VSV Kernel	SPECT VSV Kernel
Voxel Size	3 mm isotropic	0.98 mm x 0.98 mm x 3.75 mm	4.42 mm isotropic
Voxel Number	55 x 55 x 55	411 x 411 x 107	91 x 91 x 91
Materials	Water	Water	Water, Bladder, Kidney, GI, Prostate
Mass Densities (g/cm^3)	1.0	1.0	1.0, 1.03, 1.04, 1.03, 1.05
Histories/simulation	100 million	100 million	100 million

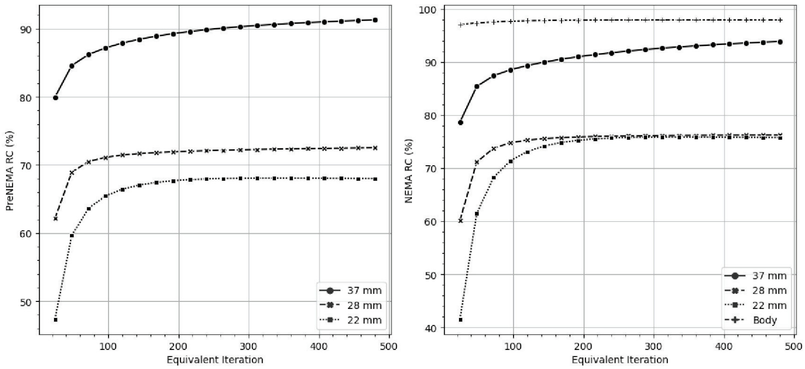

Fig. 3 The recovery coefficients of the PreNEMA and NEMA phantoms are summarized from 24 to 480 equivalent iterations.

Table 2 Recovery coefficients of the PreNEMA and NEMA phantoms

Iteration	37 mm		28 mm		22 mm		Body
	PreNEMA	NEMA2	PreNEMA	NEMA2	PreNEMA	NEMA2	NEMA2
192	87.6	91.0	68.0	75.9	60.1	75.2	97.9
480	88.7	93.8	68.8	76.2	60.2	75.8	97.9

Table 3 The percent error (%) of mean doses using VSV kernel and LDM for the PreNEMA phantom at select iterations.

Iteration	37 mm		28 mm		22 mm	
	Sphere	PVV	Sphere	PVV	Sphere	PVV
LDM at 192	-20.6	544	-37.9	675	-44.7	496
LDM at 480	-19.5	512	-37.1	657	-44.6	496
VSV at 192	-11.6	686	-23.0	825	-38.9	621
VSV at 480	-10.5	652	-30.3	806	-38.7	621

Table 4 The percent error (%) of mean doses using VSV kernel and LDM for the NEMA phantom at select iterations.

Iteration	37 mm		28 mm		22 mm		Body
	Sphere	PVV	Sphere	PVV	Sphere	PVV	
LDM at 192	-17.9	11.9	-31.0	36.5	-31.2	17.6	-18.0
LDM at 480	-15.3	1.38	-30.7	34.4	-30.7	14.1	-18.0
VSV at 192	-8.07	37.2	-23.0	63.0	-23.6	41.1	-2.0
VSV at 480	-5.24	25.6	-22.7	60.1	-23.0	37.2	-1.97

Table 5 A summary of the ground truth mean absorbed dose (Gy/MBq/s) for PreNEMA and NEMA phantom.

Iteration	37 mm		28 mm		22 mm		Body
	Sphere	PVV	Sphere	PVV	Sphere	PVV	
PreNEMA	39.0	0.18	38.9	0.13	38.5	0.10	0.01
NEMA	39.3	2.29	38.7	2.25	38.3	2.22	2.08
